# Isolated Horizontal Gaze Palsy: Observations and Explanations

**DOI:** 10.3389/fneur.2017.00611

**Published:** 2017-11-15

**Authors:** Renee Ewe, Owen B. White, Ailbhe Burke

**Affiliations:** ^1^Whittington Hospital, London, United Kingdom; ^2^Department of Neurology, Royal Melbourne Hospital, Parkville, VIC, Australia; ^3^Department of Medicine, Royal Melbourne Hospital, University of Melbourne, Melbourne, VIC, Australia; ^4^The National Hospital for Nervous Diseases, London, United Kingdom

**Keywords:** ophthalmoplegia, horizontal gaze, pontine anatomy, paramedian pontine reticular formation, median longitudinal fasciculus

## Abstract

We present three cases that we suggest require a novel diagnosis and a reconsideration of current understandings of pontine anatomy. In this case series, we highlight a series of patients with monophasic, fully recovering inflammatory lesions in the pontine tegmentum not due to any of the currently recognized causes of this syndrome. We highlight other similar cases in the literature and suggest there may be a particular epitope for an as-yet-undiscovered antibody underlying the tropism for this area. We highlight the potential harm of misdiagnosis with relapsing inflammatory or other serious diagnoses with significant adverse impact on the patient. In addition, we propose that this would support a reinterpretation of the currently accepted anatomy of the pontine gaze inputs to the median longitudinal fasciculus and paramedian pontine reticular formation.

## Introduction

Bilateral horizontal gaze palsy is a rare presentation caused by bilateral interruption of the median longitudinal fasciculus, abducens nucleus, or the paramedian pontine reticular formation (PPRF) (1, 2). The types of eye movements that may be affected in brain-stem lesions include horizontal and vertical slow eye movements, pursuit movements, vestibular and optokinetic responses, and fast eye movements, including voluntary or evoked saccades and fast phases of vestibular and optokinetic stimuli. Vergence control may be affected by midbrain lesions.

Horizontal gaze paralysis has been described as a consequence of infarction (3), inflammation/demyelination as part of multiple sclerosis (MS) or neuromyelitis optica spectrum disorders (NMOSD) (2, 4, 5), hemorrhage (6), and metastasis (7). Of the previously reported non-vascular/tumor related cases, five of six had bilateral horizontal gaze palsies and three had solitary lesions on magnetic resonance imaging (MRI), localized to the pontine tegmentum. Milea et al. (1) reported two cases of bilateral internuclear ophthalmoplegia (INO) transforming into bilateral horizontal gaze palsies with single T2-enhancing lesions in the same location with the same morphology as our case series. They suggested MS as a probable diagnosis.

Henderson described a patient with horizontal diplopia on left lateral gaze and adduction weakness (8). MRI brain demonstrated a solitary T2 hyperintense brain-stem lesion adjacent to the fourth ventricle, and a diagnosis of MS was made. In the absence of additional clinical information, it is difficult to discern how this diagnosis was made. Matsui et al. reported a patient complaining of horizontal diplopia with bilateral horizontal gaze paresis (9). A diagnosis of Bickerstaff’s brain-stem encephalitis (BBE) was made although the clinical features were inconsistent with the diagnostic criteria (10).

Here, we describe three patients with bilateral horizontal gaze paresis, caused in each case by a solitary, inflammatory lesion affecting the dorsal tegmentum of the pons extending forwards to the region of the median longitudinal fasciculus (MLF). We believe these three cases highlight questions regarding the pathogenesis of such syndromes and the relationship of horizontal and vertical gaze related projection into the MLF in particular.

As per the Ethics requirement of our institution, Royal Melbourne Hospital, informed, written consent for the use of anonymized data was obtained from all patients.

## Patient 1

A 38-year-old man of Turkish origin presented with acute intermittent diplopia, dizziness, and vomiting; he was diagnosed with peripheral vertigo and discharged. One week later, he re-presented with left-sided facial numbness. His personal medical history, systems review and family history were unremarkable.

Visual acuity was 0.63 OU, measured on the decimal scale. Pupil diameter was 5 mm bilaterally and non-reactive. Ocular motility examination (Table 1) revealed complete failure of all horizontal eye movements, voluntary saccades, smooth pursuit, vestibular, and optokinetic movements. Vertical saccades, smooth pursuit, and vestibular movements were intact to clinical examination but this does not exclude the possibility of minor slowing that would have required oculography for identification. The neurological examination was otherwise normal.

**Table 1:** Summary of the clinical oculomotor findings, MRI imaging findings and CSF results.

Patients	Patient 1	Patient 2	Patient 3

Eye movements			
**Horizontal**			
Voluntary saccades	Absent	Slowed (asymmetric)	Slowed (bilateral INO)
Smooth pursuit	Absent	Impaired	Impaired
Vestibular	Absent	Impaired	Impaired
Optokinetic	Absent	Impaired	Not reported
Nystagmus	Nil	Nil	Nil
**Vertical**			
Voluntary saccades	Normal	Normal	Slowed
Smooth pursuit	Normal	Normal	Normal
Vestibular	Normal	Normal	Not reported
Optokinetic	Not reported	Normal	Not reported
Nystagmus	Nil	Upbeat	Upbeat
**Magnetic resonance imaging**			
	Dorsal pons	Dorsal pons	Dorsal pons
	T2 hyperintense	T2 hyperintense	T2 hyperintense
	Fluid-attenuated inversion recovery (FLAIR) hyperintense	FLAIR hyperintense	FLAIR hyperintense
	DWI restriction	DWI restriction	DWI restriction
**Cerebrospinal fluid**			
	Unmatched oligoclonal immunoglobulins bands (OCBs)	Unmatched OCBs	No OCBs
	Nil else	Nil else	8 lymphocytes

**Figure 1:**
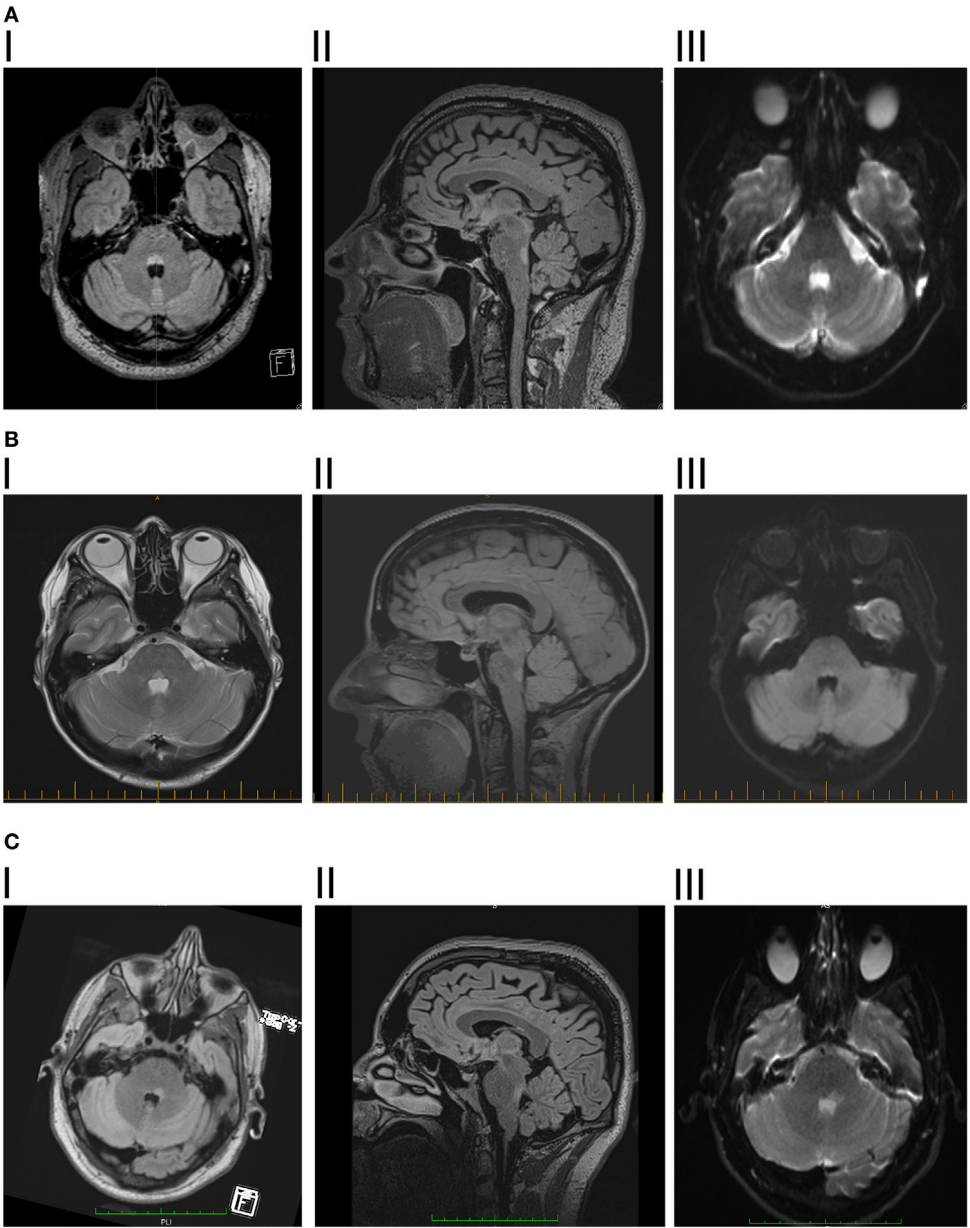
**(A)** I. Patient 1, axial fluid-attenuated inversion recovery (FLAIR) showing hyperintense lesion in posterior pontine tegmentum. II. Saggital FLAIR showing vertical extent of the lesion. III. Diffusion-weighted imaging showing mild diffusion restriction. **(B)** I. Patient 2, axial dual showing hyperintense lesion in posterior pontine tegmentum. II. Saggital FLAIR showing vertical extent of the lesion. III. Diffusion-weighted imaging showing mild diffusion restriction. **(C)** I. Patient 3, axial flair showing hyperintense lesion in posterior pontine tegmentum, slightly more significant on the right side. II. Saggital FLAIR showing vertical extent of the lesion, slightly larger than the previous two patients, consistent with there being some slowing of vertical saccades. III. Diffusion-weighted imaging showing mild diffusion restriction.

Brain MRI showed a T2 and fluid-attenuated inversion recovery (FLAIR) hyperintense triangular lesion within the dorsal pons in the floor of the fourth ventricle (Figure 1A). The lesion displayed mildly restricted diffusion matched on apparent diffusion coefficient mapping, consistent with reports in the literature as occurring with inflammatory/demyelinating lesions (11, 12), and did not exhibit contrast enhancement. MRI of the brain and spine was otherwise unremarkable. The brain-stem lesion was felt to have features consistent with demyelination although no further lesions were seen. Behçet’s disease, vasculitis, or a mitotic lesion were considered as differential diagnoses.

Cerebrospinal fluid (CSF) analysis revealed three lymphocytes, no polymorphs, and no red blood cells, oligoclonal immunoglobulins bands (OCBs) were seen in the CSF and were unmatched in serum. There was minimally elevated protein (0.46 g/l), but testing was negative for anti-aquaporin 4 (Aq4). Serum Aq4 was likewise negative. Testing for anti-ganglioside antibodies was not done and anti-myelin oligodendrocyte glycoprotein (MOG) antibodies were not available at the time of acute illness but were negative when they became available 2 years later.

The patient was treated for presumed demyelination and improved after 3 days of 1 g intravenous (IV) methylprednisolone. His neurological examination had returned to normal on review 3 weeks after discharge. Serial brain MRIs have demonstrated complete resolution of the pontine lesion, with no new lesions seen, and no further clinical episodes over 2 years since the initial presentation.

## Patient 2

A 46-year-old female presenting with frontal headache was treated for migraine and initially responded to naproxen and amitriptyline. Six days later, she re-presented with worsening headache, photophobia, blurry vision, intermittent horizontal diplopia, and fluctuating facial numbness.

Her medical history and family history were unremarkable. Neuro-ophthalmic examination revealed visual acuity of 0.32 OU. Pupillary responses were reported as slightly sluggish but reactive bilaterally. Oculomotor examination is summarized in Table 1. There was an asymmetric bilateral horizontal gaze paresis, right worse than left. Saccades to the right were restricted in amplitude and slow. Vestibular responses were asymmetric with doll’s head maneuver evoked eye movements being normal in the left eye but restricted in the right. More active head thrust responses were hypoactive bilaterally. Upbeat nystagmus was most evident on upgaze but with ophthalmoscopy was noted also in the primary position. Vertical saccades, up and down were normal. Vertical smooth pursuit appeared intact. Vertical head thrusts and optokinetic responses were clinically normal. Video oculography was not available and, overall, these findings were interpreted as being consistent with mild impairment of vertical gaze. The vertical nystagmus resolved in 2 days. She also had mild bilateral facial weakness, absent tendon reflexes in the upper limbs, and mild gait ataxia. The clinical impression was that her constellation of symptoms was consistent with Miller Fisher Syndrome, BBE or central demyelination, but without evidence to confirm the diagnosis of these entities on testing.

Magnetic resonance imaging brain revealed a solitary, well-defined paramedian focus of T2 hyperintensity in the dorsal pons which displayed mildly restricted diffusion but no contrast enhancement (Figure 1B). MRI spine was normal.

Nerve conduction studies revealed bilateral facial motor axonal neuropathy. CSF analysis demonstrated OCBs (unmatched) and elevated albumin (298 mg/l). Serum Aq4 antibody was negative. Routine CSF results were normal, including protein 0.4 g/l, white cell count (WCC), and red cell count (RCC) < 1. CSF was negative for anti-AQP4, anti-GM1, anti-GQ1B, and anti-myelin-oligodendrocyte protein antibodies.

The diagnosis was one of a localized encephalitis restricted to the pons and primarily affecting dorsal tegmentum. The patient was treated with a 5-day course of 2 g/kg IV immunoglobulin. Over the next week, ophthalmoplegia improved significantly, upper limb reflexes improved and ataxia resolved. No new symptoms or lesions were identified over 12 months of follow-up. Review at 18 months confirmed complete clinical recovery.

## Patient 3

This 29-year-old man woke with sweats and chills, followed by bifrontal headache, postural vertigo, nausea, and vomiting. At presentation 12 h later, he had horizontal diplopia and right-sided numbness. He disclosed a 5-day history of upper respiratory tract symptoms, blocked sinuses, and a pertussis immunization booster 1 month prior to symptom onset. His history included hypertension, hypothyroidism, anxiety, irritable bowel syndrome, obesity, and non-alcoholic steatohepatitis.

Magnetic resonance imaging brain revealed a left-sided non-enhancing T2 hyperintense dorsal pontine lesion, which demonstrated restricted diffusion (Figure 1C). CSF was remarkable for elevated WCC of 8 × 10^6^/l (7 lymphocytes, 1 neutrophil), RCC was 4 × 10^6^/l and CSF protein was 0.35 g/l. This was initially diagnosed as ischemic stroke, at another institution, and medical therapy for secondary prevention was started. No abnormalities were found on magnetic resonance angiography, cardiac monitoring, transesophageal echocardiogram, vasculitis, and thrombophilia screens.

Two weeks after onset he developed altered left-sided taste, left tongue, inner mouth, and facial numbness. His monocular visual acuity was 1.0 OU, but dynamic visual acuity was 0.4 OU. Pupils responses were normal. He demonstrated bilateral gaze paresis and INO (Table 1). Head thrust responses were bilaterally hypoactive with mildly slowed upward saccades and upbeat nystagmus on upgaze. The rest of the neurological examination revealed mild left dysmetria only.

Repeat CSF analysis revealed 3 leukocytes (undifferentiated), 27 red cells, and negative for OCBs. Non-contrast MRI Brain revealed enlargement of the lesion with persistent restricted diffusion. The appearances were reported consistent with demyelination. Serum anti-ganglioside antibodies, anti-aquaporin-4 antibody (AQP4), and anti-MOG antibodies were negative.

He was treated with a 3-day course of 1 g daily IV methylprednisolone followed by 60 mg oral prednisolone daily, weaned slowly after 1 month. A follow-up examination 8 weeks after first presentation demonstrated a residual left INO and right sensory symptoms only, and these resolved over subsequent months. The lesion resolved completely on follow-up imaging 3 months later.

## Discussion

We describe three cases of bilateral horizontal gaze paresis, coincident with the acute development of a small, well-circumscribed lesion of the pontine tegmentum on MRI. By definition, these cases represent clinically isolated syndromes but in the absence of recurrent lesions in 12–36 months of follow-up. The phenotype is described in MS, NMOSD, stroke, and tumor (1, 4, 5, 8, 9, 13, 14). However, patients with these diagnoses tend to develop relapses and characteristic MRI changes (4, 15). In our cases, there is insufficient evidence for any of these diagnoses at presentation or on follow-up. We focus here on the proposed clinical and anatomical novelties they demonstrate.

Anti-GQ1b spectrum disorders can affect ocular movements and could potentially present with the clinical features we described. Miller–Fisher/BBE overlap syndrome was considered in the second case but these patients tend to present with positive anti-GQ1B antibodies and normal MRI brain (13). As such, we feel an alternative diagnosis is more likely. Although NMOSD can present with inflammatory lesions near the pontine tegmentum, they are characteristically slightly lower at the floor of the fourth ventricle. These cases reported here do not fulfill the recently updated criteria for NMOSD (16).

Similarly, CLIPPERS syndrome is a brain-stem inflammatory disorder but is clinically and radiologically more diffuse and unlikely to be an explanation in our patients (17).

Control of vertical gaze is generally ascribed to the rostral midbrain, the rostral interstitial nucleus of the MLF (riMLF) containing burst cells, and the interstitial nucleus of Cajal forming the neural integrator for maintenance of position in the orbit. These regions project bilaterally, partly *via* the posterior commissure. Inputs to these areas derive from a number of sources, partly dependent on the type of input.

Vestibular inputs derive from the superior vestibular nuclei bilaterally, while smooth pursuit signals project from the Y group of cells to the riMLF. Pathways include the MLF, ventral tegmental tract, and cerebellar pathways. Voluntary and reflex vertical saccades, determined by supratentorial networks, appear to be dependent on projections from the PPRF region bilaterally, and are abolished by localized lesions of the caudal PPRF bilaterally only (18). A strong omnipause neuron projection has been demonstrated from the PPRF, bilaterally, to the riMLF (19, 20). These must be inhibited for voluntary and reflexive saccades, not fast phases, to be generated (21).

Vertical gaze may be spared in the presence of horizontal ophthalmoplegia with a posterior pontine tegmental lesion with or without more widespread pathology (1, 13). Although pure abducens lesions may produce horizontal gaze palsy, the cases reported by Milea et al. presented early with bilateral INO, suggestive of involvement of either the MLF or bilateral projections into the MLF.

The unifying lesion, in our patients and those referenced here, is the posterior tegmental lesion. This lesion is in close proximity to the abducens nuclei and to the PPRF containing the excitatory burst cells for horizontal gaze. Bilateral involvement of projections from the PPRF or the abducens nuclei would explain horizontal gaze palsy. Relative sparing of vertical eye movements implies that the bilateral vertical signals projecting to the mesencephalic reticular formation vertical gaze center *via* the MLF (22), run a distinct pathway in the pons, compared to the lateral gaze inputs. This is consistent with evidence from Pierrot-Deseilligny et al. (7), on dissociated bilateral horizontal gaze paralysis, but at odds with the current conventional clinical perception of anatomy in this area.

If there are bilateral lesions of the lateral gaze centers in the PPRF, closely associated with and almost indistinguishable from the abducens nucleus, conventional wisdom would indicate that there should be vertical gaze palsies also as those pathways are said to run together. Certainly, midline lesions affecting both median longitudinal fasciculi (MLF) produce bilateral INO which is characteristically associated with vertical gaze paresis, characterized by impaired vertical smooth pursuit and vestibulo-ocular reflex cancellation (23). More rostral lesions may affect midbrain structures (riMLF and INC) involved in vertical saccades (24). No such vertical gaze impairment is seen in our patients.

We suggest that the vertical and horizontal gaze pathways run separately through their lower course and merge in their rostral course, with the vertical gaze inputs running anterolateral to the horizontal gaze inputs. They may cross in their ascent, probably substantially, such that bilateral lesions are required to produce vertical gaze palsy. This would explain the sparing of the direct pathway for vertical gaze from the lateral PPRF in the presence of a unilateral lesion, abolition of vertical eye movements with bilateral localized lesions, and the requirement of bilateral stimulation to provoke vertical eye movements (18, 22).

**Figure 2:**
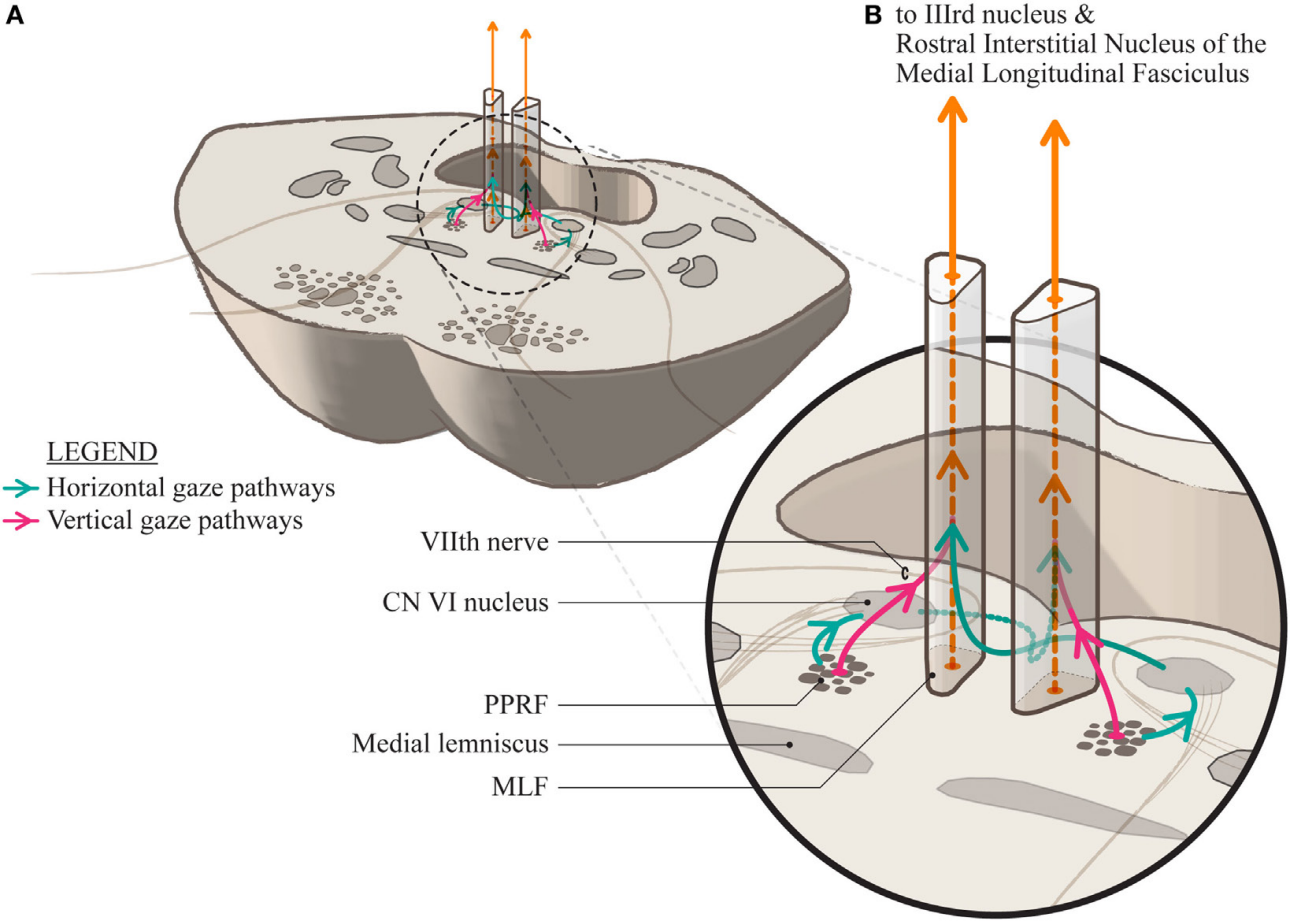
**(A)** A section through the mid-pons showing the relationship of the paramedian pontine reticular formation to the median longitudinal fasciculus (MLF) and the adjacent seventh cranial nerve as it sweeps around the abducens nucleus. **(B)** A magnification showing the proposed conformation of the separate horizontal and vertical gaze paths as they project into the MLF. The horizontal signal decussates at the level of the pons and ascends in the contralateral MLF while it is uncertain whether the vertical gaze signal decussates at all, or is a direct projection.

We propose a slightly altered anatomical arrangement to represent this (Figure 2). The horizontal gaze pathways project to the ipsilateral abducens nucleus and then ascend in the MLF to the contralateral medial rectus subnucleus of the third nerve nucleus. We propose that the vertical gaze signal projects more anteriorly into the MLF and, at least over a short distance, is separable from the horizontal gaze fiber pathways.

The similarities in the phenotype and the morphology of the causal lesions in these cases suggest that this region of the pontine tegmentum may have particular characteristics rendering it susceptible to immunological attack. None of these cases fulfill criteria for other diseases, inflammatory or otherwise, associated with MLF involvement.

If indeed these cases represent a novel phenotype, then there is a need to discern the optimal course of management, both during the acute phase and in the longer term. Misdiagnosis, for example with MS or stroke, risks exposing the patient to unnecessary long-term anti-platelet or immune-modulatory treatment and anxiety around a diagnosis of a significant, potentially recurring long-term condition with associated adverse impact on their life choices and options.

Identification of other cases of this phenotype would be useful to facilitate a search for specific new antibodies that may be at play here and to elucidate the most appropriate and effective treatment course.

## Author Contributions

RE reviewed the patient files and imaging, collected the data, and constructed the preliminary presentations as well as contributing to ongoing editing of the final submitted manuscript. OW supervised RE in the collection of the data and was involved in all levels of writing and interpreting the data as well as reviewing and editing the final manuscript hereunder. AB worked with RE in the analysis of the data, the review of the relevant literature, the interpretation of the findings and the preparation of the manuscript.

## Conflict of Interest Statement

This research was not supported financially by any commercial interests. There are no financial relationships that could be construed as a potential conflict of interest.
